# Diclofenac Alters the Cell Cycle Progression of the Green Alga *Chlamydomonas reinhardtii*

**DOI:** 10.3390/cells10081936

**Published:** 2021-07-30

**Authors:** Darya Harshkova, Ivan Liakh, Vitali Bialevich, Kamila Ondrejmišková, Anna Aksmann, Kateřina Bišová

**Affiliations:** 1 Department of Plant Physiology and Biotechnology, Faculty of Biology, University of Gdansk, Wita Stwosza 59, 80-308 Gdańsk, Poland; anna.aksmann@ug.edu.pl; 2 Department of Toxicology, Medical University of Gdańsk, Al. Gen. J. Haller 107, 80-416 Gdańsk, Poland; liakh_ivan@mail.ru; 3 Laboratory of Cell Cycles of Algae, Centre Algatech, Institute of Microbiology, Czech Academy of Sciences, Novohradská 237, 379-01 Trebon, Czech Republic; bialevich@alga.cz (V.B.); ondrejmiskova@alga.cz (K.O.); bisova@alga.cz (K.B.)

**Keywords:** non-steroidal anti-inflammatory drug, diclofenac, cell cycle, *Chlamydomonas reinhardtii*

## Abstract

The aim of the study was to verify the hypothesis that a potential cause of the phytotoxicity of diclofenac (DCF, a non-steroidal anti-inflammatory drug) is an effect of cell cycle progression. This research was conducted using synchronous cultures of a model organism, green alga *Chlamydomonas reinhardtii*. The project examined DCF effects on selected parameters that characterize cell cycle progression, such as cell size, attainment of commitment points, DNA replication, number of nuclei formed during cells division and morphology of cells in consecutive stages of the cell cycle, together with the physiological and biochemical parameters of algae cells at different stages. We demonstrated that individual cell growth remained unaffected, whereas cell division was delayed in the DCF-treated groups grown in continuous light conditions, and the number of daughter cells from a single cell decreased. Thus, the cell cycle progression is a target affected by DCF, which has a similar anti-proliferative effect on mammalian cells.

## 1. Introduction

Algae are a basic element of the water environment, and thus any abnormality in their function may lead to a disruption in the stability of a particular ecosystem. Hence, it is important to understand how different toxic substances influence algal physiology and biochemistry. Diclofenac (DCF), belonging to the non-steroidal anti-inflammatory (NSAID) class of drugs, is one of the pharmaceuticals on the hazardous substances list [1] and one of the most common pollutants of water environments [2]. As with other pharmaceuticals, DCF is designed to influence specific metabolic pathways in animal and human cells. Thus, ecotoxicological research is mostly focused on the threat DCF poses to non-target animal organisms [3]. In this regard, some investigations into DCF phytotoxicity mainly describe general observations. For example, it was shown that DCF inhibits the growth of duckweed *Lemna minor* (Magnoliophyta) as well as the reproduction of green algae *Scenedesmus vacuolatus*, *Desmodesmus subspicatus* and *Dunaliella tertiolecta* (Chlorophyta) [4,5,6,7]. Furthermore, DCF was found to be phytotoxic in standard tests as defined by the OECD or ISO guidelines [8].

In our earlier studies, *Chlamydomonas reinhardtii* (*C. reinhardtii*) population growth was found to be suppressed by DCF [9], which could have resulted from either cell growth inhibition or cell division blockage. However, when using an asynchronous culture, it is impossible to separate the two; thus, the cause of the abovementioned effects at the biochemical and molecular level remained unresolved. Here, we attempt to fill this knowledge gap by examining the effect of DCF on the growth and cell cycle progression using synchronous algal cultures. The alga *C. reinhardtii* is a suitable model organism for the study as it has previously been exploited for both toxicity testing and analyses of the cell cycle [9,10,11,12]. Furthermore, depending on growth conditions, *C. reinhardtii* will divide into 2 or more daughter cells through a mechanism called multiple fission [13]. Its cell cycle is composed of several non-overlapping growth sequences that are coordinated with overlapping reproductive sequences. Each reproductive sequence starts with the attainment of a commitment point (CP), followed by DNA replication (S phase), nuclear division (M phase) and cell division (C phase) [13,14,15,16,17]. The attainment of a CP is dependent on reaching a specific, critical cell size [17,18], which interconnects both growth and reproductive sequences. At sufficiently fast growth rates, more than one CP, in principle *n*, can be attained. Each of these will eventually lead to completion of one reproductive sequence [19] independent of further energy, even in the dark. This way, the mother cell can divide into 2, 4, 8 or 16 daughter cells, in principle to 2*n* cells [13]. The reproductive events are more sensitive to potentially toxic compounds than cell growth [20]. Impairment of DNA replication or nuclear or cellular division may result in a partial blocking or delaying of one or more of these. The order of sensitivity of the reproductive sequence is opposite to the order of performance within the cell cycle, with cell division being the most sensitive and DNA replication the least sensitive. The application of a toxic compound is often manifested by partial or complete blockage of cell division or a lower number of daughter cells being formed that do not correspond to the number of CPs attained.

Here, we used synchronous cultures of *C. reinhardtii* to describe in detail the influence DCF has on the algal cell cycle and discriminate it from its effect on growth. In particular, we studied whether the growth inhibition observed in asynchronous cultures resulted from inhibiting the growth of individual cells or from inhibiting cell division. Furthermore, we were interested in whether the mode of action of DCF differed with changes in its concentration and how DCF affected the timing and extent of DNA replication and nuclear and cellular divisions.

## 2. Materials and Methods

### 2.1. Culture Conditions and Exposure to Toxicant

*C. reinhardtii* 21 gr (CC–1690, wild type; Chlamydomonas Resource Center, St. Paul, Minnesota, USA, http://www.chlamy.org, accessed on 27 July 2021) was grown in glass vessels in a mineral medium (HSM) [21], at 30 °C, with air-CO_2_ (2% *v*/*v*) aeration. The vessels were illuminated by fluorescent tubes (OSRAM DULUX L55 W/950 Daylight, Milano, Italy) and the light intensity at the culture vessel surface was 500 µmol·m^−2^·s^−1^ of photosynthetically active radiation (PAR). To synchronize population growth, alternating cycles of 11 h light and 13 h dark were applied [12] to the cultures being diluted to a constant cell number at the beginning of each light period. At the beginning of the experiment, the synchronized cell population was diluted to a starting cell concentration between 4.5 × 10^6^ and 4.7 × 10^6^ cell/mL and divided into five sub-populations: one control and four DCF-treated variants. DCF was dissolved in ddH_2_O and added to cells cultures at the beginning of the light period (0 h) to obtain a final concentration of 32.7, 65.75, 135.5 and 203.25 mg/L, corresponding to the EC10/24, EC25/24, EC50/24 and EC75/24, respectively, as previously described [9]. The abovementioned subpopulations are hereafter described as control, DCF10, DCF25, DCF50, and DCF75, respectively. All subpopulations were then grown under continuous light (500 µmol photons m^−2^ s^−1^ of PAR, 30 °C, 2% *v*/*v* CO_2_) and sampled in the same way. Samples of algae growth were taken every hour from 0 h to 19 h for further analyses. In the DCF75 culture, high cell mortality was observed in the microscopic samples as the absence of chlorophyll auto-fluorescence when comparing the light and fluorescence microscopy of the same sample. Very soon, the dead cells lysed and a sharp decline in cell numbers was observed. This led to over 70% mortality of the population within the first hours of the cell cycle; further analyses were not continued at this condition.

### 2.2. Determination of Starch Content

Starch content was determined by the anthrone method [22] as modified by Brányiková et al. [23]. Briefly, 10 mL of algae sample was harvested by centrifugation at 3784 g for 5 min, transferred to microtubes and the cell pellet was stored at −20 °C. Defrosted samples were supplemented with 500 μL of dH_2_O and 250 μL zirconium beads (diameter 0.7 mm) and vortexed for 5 min at 3200 rpm (Vortex–Genie 2, Scientific Industries Inc., New York, NY, USA). For sample depigmentation, 1 mL of 80% EtOH was added, and the samples were vortexed and incubated in a water bath at 68 °C for 15 min. The samples were centrifuged at 24,000× *g* for 2 min and the supernatant was discarded. A sample depigmentation procedure was repeated 3–4 times until the supernatant was decolored (white or grey). Starch in the depigmented samples was hydrolyzed with 1.5 mL 30% HClO_4_ at room temperature for 15 min; then the samples were centrifuged at 24,000 g for 2 min and the supernatant was transferred to pre-prepared calibrated test tubes. Hydrolysis was repeated two more times, calibrated test tubes were topped with 30% HClO_4_ up to 5 mL and mixed by inversion using a stopper. For the colorimetric reaction, 5 mg of anthron in 2.5 mL of ice-cold 72% sulfuric acid was added to 500 μL of supernatant kept on ice, vortexed, boiled for 8 min and immediately cooled on ice. The same was done for the blank (500 μL of 30% HClO_4_) and standard tubes (500 μL of glucose (100 mg/L) in 30% HClO_4_). Spectrophotometric measurement was done at 625 nm (A_625_) using Shimadzu UV-spectrophotometer UV-1800 (UV-1800, Shimadzu, Kyoto, Japan). Calibration was carried out simultaneously using glucose as the standard. The values measured for glucose were multiplied by 0.9 to obtain a calibration curve for starch determination. The data are expressed in pg of starch content per cell or in ag per µm^3^ of total volume of biomass in the sample.

### 2.3. Photosynthetic Pigment Contents in the Cells

Photosynthetic pigments were analyzed as described in Řezanka, et al. [24]. Briefly, 10 mL of algae sample were harvested by centrifugation for 3 min at 3784 g, and the cell pellet was supplemented with 1 mL of phosphate buffer (pH 7.7) containing a small amount of MgCO_3_ to stabilize the chlorophylls and stored at −20°C until used. Cells in defrosted samples were disintegrated with 500 µL of zirconium beads (diameter 0.7 mm) by vortexing for 10 min at 3200 rpm (Vortex-Genie 2, Scientific Industries Inc., New York, NY, USA). The chlorophylls were extracted with 4 mL of 100% acetone followed by centrifugation for 3 min at 3784× *g*. The supernatant was transferred into calibrated test tubes which were closed with a stopper and put in a dark block. The extraction step was repeated with 4 mL of 80% (*v*/*v*) acetone and the volume of supernatant was topped to 10 mL with 80% (*v*/*v*) acetone. For analyzing chlorophyll content in the algal suspension, the samples were measured on a UV–Vis spectrophotometer (UV-1800, Shimadzu, Kyoto, Japan) in 1 cm cuvettes at 750, 664, 647, 470, 450 nm with 80% acetone as a blank. Calculation of chlorophyll content from absorbance measured at 645 and 664 nm was carried out using the method from MacKinney [25]. The following equations were used for Chlorophyll *a* (chl *a*) (12.25 × A664 nm–2.79 × A647 nm), Chlorophyll *b* (chl *b*) (21.5 × A647 nm−5.1 × A664 nm) and carotenoids ((1000 × A470 nm)−(1.82 × chl *a*)−(85.02 × chl *b*))/198) [25,26,27].

### 2.4. Chlorophyll a Fluorescence

Chlorophyll *a* fluorescence (OJIP test) in vivo was measured using an AquaPen AP100 fluorometer (Photon System Instrument, Drásov, Czech Republic) using FluorPen 1.1.2.3 software. The following OJIP parameters were analyzed: QY–the quantum yield, PI–the performance index, (an overall expression indicating algal “vitality”), φPo (Fv/Fm)–the maximum yield of primary photochemistry, φE_0_–the maximum yield of electron transport, ψ0–the efficiency of moving the electron by the trapped exciton further than quinone Qa [28].

### 2.5. Cell Cycle Analyses

The attainment of CPs was assessed in hourly samples [29]. Aliquots (10 mL) were aerated at 30 °C in the dark until hour 10 and then 1 mL aliquots of the culture were placed on HSM plates and incubated in the dark at 30 °C until cell division was completed in the culture with the slowest rate of division. The plates were then examined directly under a light microscope (magnification 10 × 40). For each sample, the number of large single undivided mother cells and mother cells divided into 2, 4, or 8 daughter cells (at least 150 mother cells) were counted and the percentage of each category was calculated and plotted against time. Mother and daughter cells can be easily distinguished by differences in cell size, and cell clumps can be distinguished by the specific morphology of the divided mother cells.

Cell division was assessed in samples that were taken hourly, fixed with glutaraldehyde (final concentration 0.2%) and observed under a light microscope (magnification 10 × 40). The percentage of undivided mother cells or mother cells divided into 2, 4, or 8 daughter cells were calculated and plotted against time. Cell culture doubling time (*Td*) was calculated using the formula:

(1) $$Td=\left(tx-t_{0}\right)\frac{\ln\left(2\right)}{\ln\left(\frac{N_{x}}{N_{0}}\right)}$$

where *N*_0_ is the number of cells at the beginning of the experiment (*t*_0_), and *N_x_* is the number of cells at the end (*t_x_*) of the experiment [30]. The data are expressed in numbers of hours. Additionally, the division number was calculated using the formula: *N_x_*/*N*_0_, with *N*_0_ and *N_x_* being the number of cells at the start (*t*_0_) and end (*t_x_*) of the experiment.

### 2.6. Cell Volume

Cell volumes were measured with the Multisizer 4 (Beckman Coulter) in glutaraldehyde-fixed samples (final concentration 0.2%) by diluting 50 µL of fixed cells in 10 mL 0.9% NaCl. The results were given as modal volume values of the cells in the sample. For determining daughter cell size, 10 mL aliquots from 9 h and 11 h of the cell cycle were aerated at 30 °C in the dark until division was complete (not more than 24 h from start of illumination), samples were fixed with gluteraldehyde and analyzed.

### 2.7. Nucleic Acid Analyses

Total nucleic acids were extracted according to Wanka (1962) [31], as modified by Decallonne and Weyns (1976) [32]. The DNA test was performed as described by Decallonne and Weyns (1976) [32], with modifications by Zachleder (1984) [33]. The sediment remaining after nucleic acid extraction was quantified for protein content according to the procedure described by Lowry and Randall (1951) [34]. The data are expressed in pg of DNA, RNA or protein content per cell or in fg per µm^3^ of total volume of biomass in the sample.

### 2.8. Microscopy Observation and 4′,6-Diamidine-2′-Phenylindole Dihydrochloride Staining

Cell nuclei were stained with 4′,6-diamidine-2′-phenylindole dihydrochloride (DAPI). Samples from the culture (1 mL) were centrifuged at 1960 g for 3 min; the supernatant was removed; and samples were kept at −20 °C. Before analysis, 10 μL of DAPI solution (5 μg/mL DAPI in 0.25% (*w*/*v*) sucrose, 1 mM EDTA, 0.6 mM spermidine, 0.05% (*v*/*v*) mercaptoethanol, 10 mM Tris–HCl, pH 7.6) was added to the frozen samples. The samples were then mixed and incubated for 20 min in the dark at room temperature. The number of nuclei in the mother cells was calculated using observations from transmitted light and fluorescence microscopy carried out using an Olympus BX51 microscope (Olympus Czech Group, Prague, Czech Republic) equipped with a CCD camera (DP72). AU-MWIBA2 filter block (Life Technologies, Prague, Czech Republic) (Ex/Em: 360–370/420–460 nm) was used for DAPI fluorescence, chlorophyll autofluorescence was detected as a red signal [29].

### 2.9. Statistical Analyses

Statistical analyses, including the basic ones, were carried out using MS Excel 2010 (Microsoft) using Real Statistics Resource Pack (https://www.real-statistics.com/free-download/real-statistics-resource-pack/, accessed on 27 July 2021) and Statistica 9.0 (StatSoft, Tulsa, OK, USA). All data were expressed as the mean of at least three independent experiments (biological replicates) ± SE. As the populations could not be assumed to be normally distributed, a comparison of correlation coefficients was used to analyze the difference between the groups. After establishing correlation coefficients for each group, they were transformed to *Z* values using the Fisher transformation. The difference between the *Z* values was analyzed using a *t*-test. Tukey’s HSD–Repeated Measures ANOVA test was used to compare the final percentage of cells that attained individual CPs or cell division and nonparametric Mann–Whitney U tests. A *p*-value < 0.05 was considered significant.

## 3. Results

### 3.1. Analysis of Cell Size

**Figure 1:**
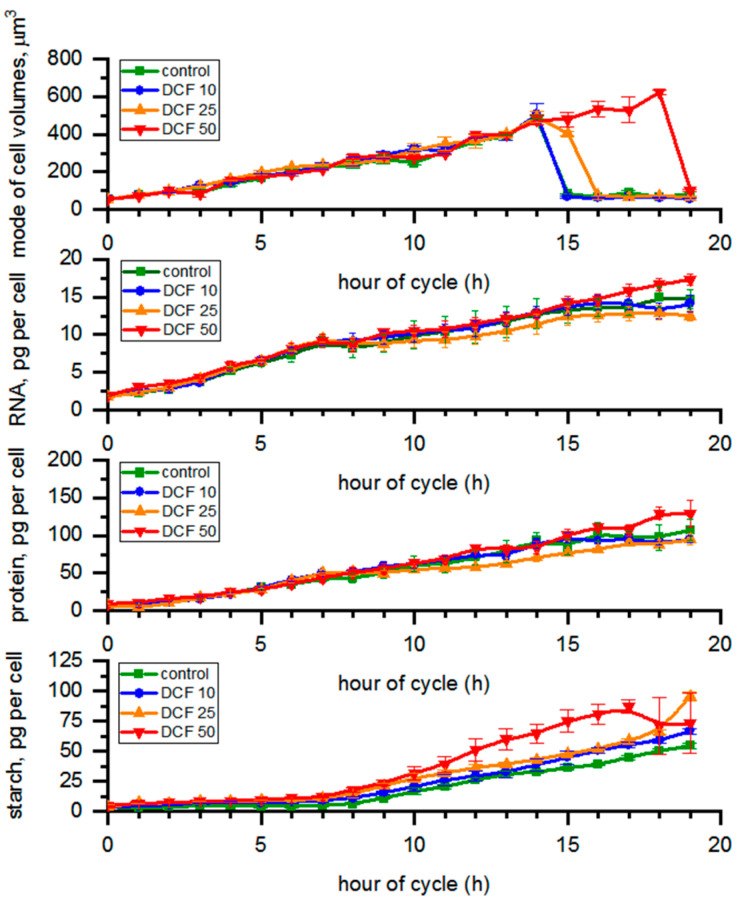
Cell volume, RNA, protein, and starch content calculated per single mother cell. Data are presented as means ± SE. The mode cell volume and starch content of DCF50 were statistically significantly different from controls (correlation comparison, *p* < 0.05, *n* = 3). Data are presented as pg per mother cells even at the time of cell division to allow simple comparison across the groups.

The cell volumes in control, DCF10 and DCF25 ranged between 56 µm^3^ (young daughter cells at the beginning of the cell cycle) and 490 µm^3^ (mature mother cells at the 14th h) during the cell cycle (Figure 1). However, the liberation of daughter cells in DCF25 was delayed by 2 h compared to control. DCF50 cells continued growth until 19 h and mature mother cells reached approx. 600 µm^3^ before daughter cell liberation began.

### 3.2. RNA Content and Protein Accumulation

The content of all biomolecules (RNA, protein, starch, photosynthetic pigments, DNA) was expressed per single mother cell. This held true even at the time of cell division; thus, the presented values were not affected by undergoing cell division. We chose this normalization because plotting the RNA and protein per cell during the time when the entire population was dividing into two and four cells would generate a very large error and be hard to interpret reliably. The RNA content in the control changed from 1.75 to 14.99 pg per cell during the cycle (Figure 1), while protein content varied from 7.62 to 107.28 pg per cell. The RNA and protein content in single cells did not change significantly in any treated cells compared to control (Figure 1). From the 14 h samples, cell volume in different experimental groups varied from each other (Figure 1). We also re-calculated the total RNA and protein content per µm^3^ of total biomass volume in each sample (Figure S1). The RNA content in the control samples changed from 27.08 to 47.23 fg per µm^3^ of biomass volume (Figure S1). In all DCF variants, the RNA content in the biomass tended to decrease with time compared to controls, and the differences were statistically significant for DCF25 and DCF50 (Figure S1). Protein content in controls varied from 0.15 to 0.33 pg per µm^3^ of biomass volume (Figure S1), and it followed a similar trend to RNA content but the difference was statistically significant only for DCF50.

### 3.3. Analysis of Starch Content in the Cells

The starch content in controls varied from 3.92 to 54.50 pg per cell during the course of the cycle (Figure 1). The starch content in DCF50 cells tended to increase compared with control, and this trend was statistically significant. Recalculation of the starch content to per µm^3^ of total biomass volume in samples (Figure S1) revealed the same trend in DCF50, while in DCF10 and DCF25 the starch content was similar to that of the control.

### 3.4. Analysis of Photosynthetic Pigments Content

**Figure 2:**
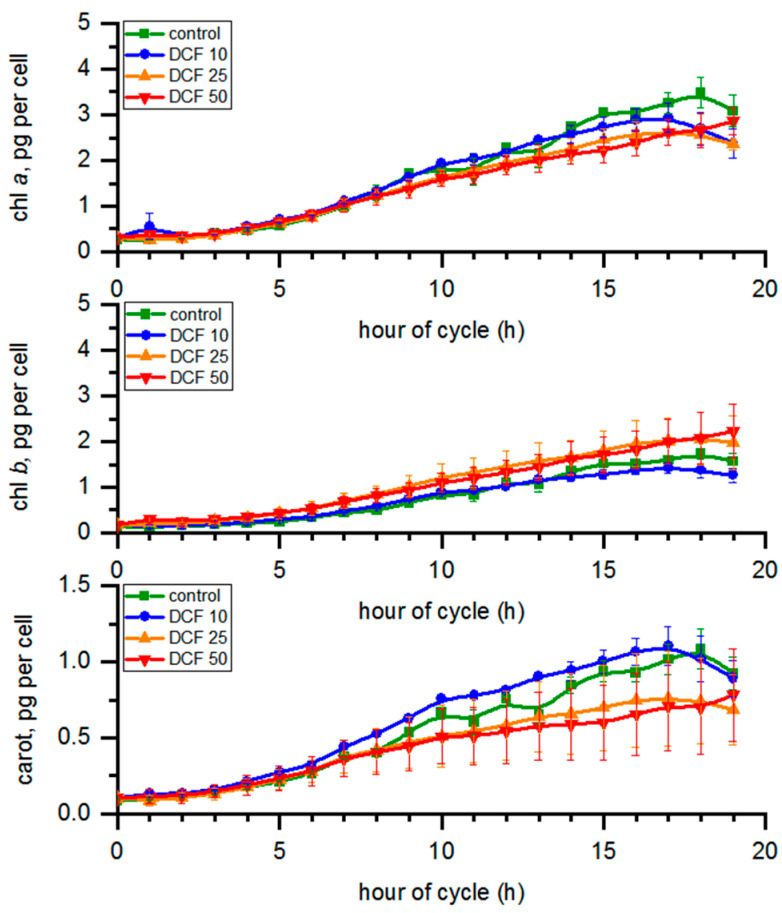
The photosynthetic pigment content calculated per single mother cell. Data are presented as means ± SE. Chlorophyll *b* and carotenoid content of DCF25 were statistically significantly different from the controls. Chlorophyll *a*, chlorophyll *b* and the carotenoid content of DCF50 were statistically significantly different from controls (correlation comparison, *p* < 0.05, *n* = 3). Data are presented as pg per mother cells even at the time of cell division to allow simple comparison across the groups.

During the course of the cycle (Figure 2), the chl *a* content in the control changed from 0.26 to 3.49 pg per cell; for chl *b*, the concentration ranged from 0.15 to 1.73 and carotenoid content ranged from 0.09 to 1.08 pg per cell (Figure 2). Chl *a* content in all DCF-treated cells tended to decrease, with the most pronounced effect in DCF50 where it was statistically significant. The level of chl *b* significantly increased (*p* ≤ 0.05) in DCF25 and DCF50 cells. The carotenoid content in DCF25 and DCF50 cells was significantly lower, while in DCF10 cells it was slightly higher compared with control (Figure 2). The same trend was visible after recalculation of the photosynthetic pigment content per µm^3^ of total biomass volume in the sample (data not shown).

### 3.5. Analysis of Photosynthetic Parameters by OJIP Method

**Figure 3:**
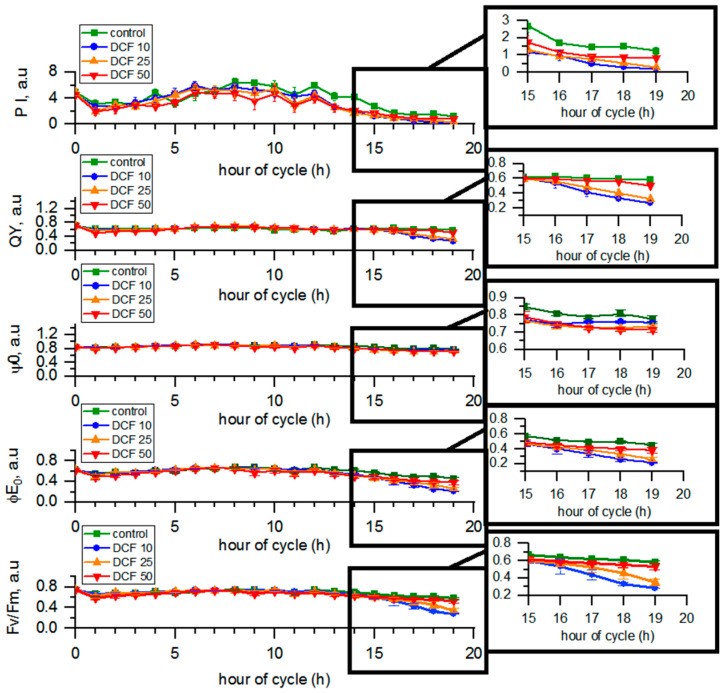
The parameters of photosynthesis obtained from the OJIP method. Data are presented as means ± SE. All the photosynthetic parameters of DCF10 and DCF25 and ψ0 of DCF50 were statistically significantly different from controls (correlation comparison, *p* < 0.05, *n* = 3).

The quantum yield (QY) and performance index (PI) values in the control group varied from 0.70 to 0.58 a.u. and from 4.87 to 1.24 a.u., respectively, during the cell cycle. The maximum yield of primary photochemistry (Fv/Fm) and the maximum yield of electron transport (φE_0_) in the control changed from 0.74 to 0.58 a.u. and from 0.62 to 0.45 a.u. during the course of the cycle, respectively. The efficiency of moving the electron by the trapped exciton further than quinone Qa (ψ0) in the control changed from 0.84 to 0.77 a.u. during the cell cycle (Figure 3). In DCF10- and DCF25-treated cells, all photosynthetic parameters statistically significantly decreased compared to controls. Surprisingly, the most pronounced effect was observed for the DCF10 variant. In contrast, photosynthesis of the DCF50-treated cells did not seem to be affected except the functioning of quinone Qa (ψ0).

### 3.6. Cell Cycle Progression

**Figure 4:**
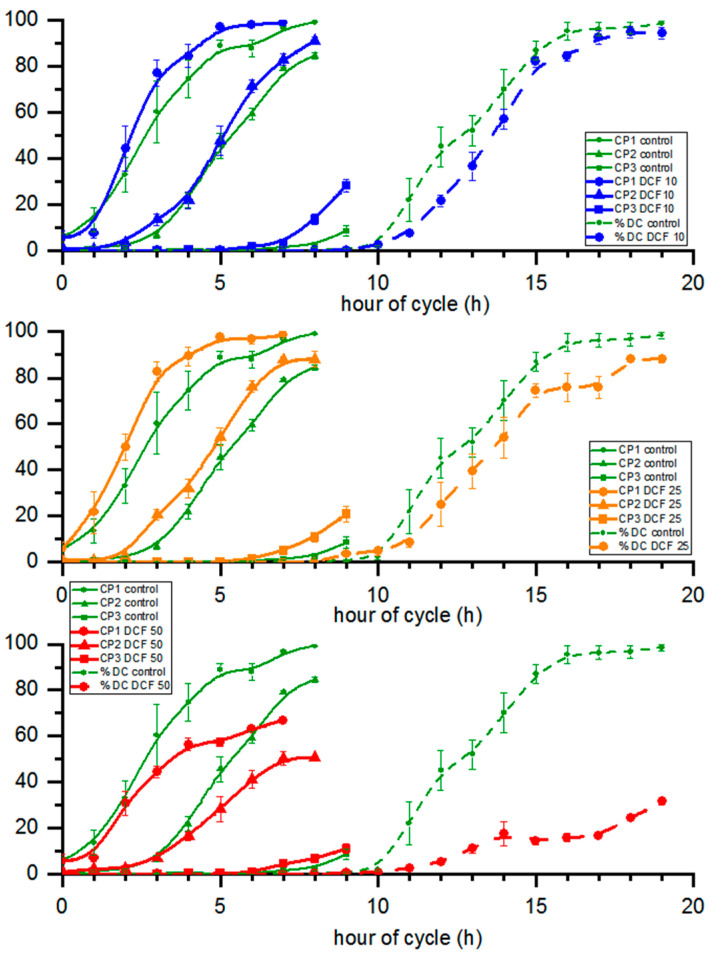
The commitment points and percent of daughter cells of cultures. Data are presented as means ± SE. Attainment of CP1, CP2 and progression of cell division of DCF50 and attainment of CP3 of DCF10 were statistically significantly different from controls (Tukey’s HSD–Repeated Measures ANOVA, *p* < 0.05, *n* = 3).

The attainment of the first, second and third CPs (CP1, CP2 and CP3, respectively) was not affected in DCF10 and DCF25 compared to the control, with the exception of CP3 in the DCF10 group, which was statistically significantly accelerated. Although the differences were not significant, all CPs were advanced by approx. half an hour in DCF10 and by 1.5 h in DCF25 (Figure 4). In contrast, the DCF50 group displayed a significant delay in CP1 and CP2. Furthermore, cell division was delayed in DCF50-treated populations compared to the control (Figure 4). Moreover, in this variant there was a significant reduction in the number of dividing cells as less than half of cells divided (Figure 4).

Apart from the changes in cell division timing, there were also differences in the number of formed daughter cells during the cycle. The division number, i.e., the average number of daughter cells formed from a single mother cell, decreased compared to the control. This was most pronounced in DCF50 with a 47% decrease but less so for DCF10 and 25 with a 27 and, 28% decrease, respectively (Table 1). Furthermore, the cell culture doubling time was prolonged in all DCF-treated variants compared to the control, increasing by 21, 21 and 54% in DCF10, 25 and 50, respectively.

While all concentrations of DCF affected the doubling time of cultures, in DCF10 and DCF25 no effect on the size of mother cells (mode cell volume) was observed. However, in DCF50 the mother cell size significantly increased (616.2 compared to 461.3 μm^3^ in the control). The size of the daughter cells increased only in DCF25 and was 51.2 compared to 39.9 μm^3^ in the control, i.e., it increased by about 28% (Table 1).

**Figure 5:**
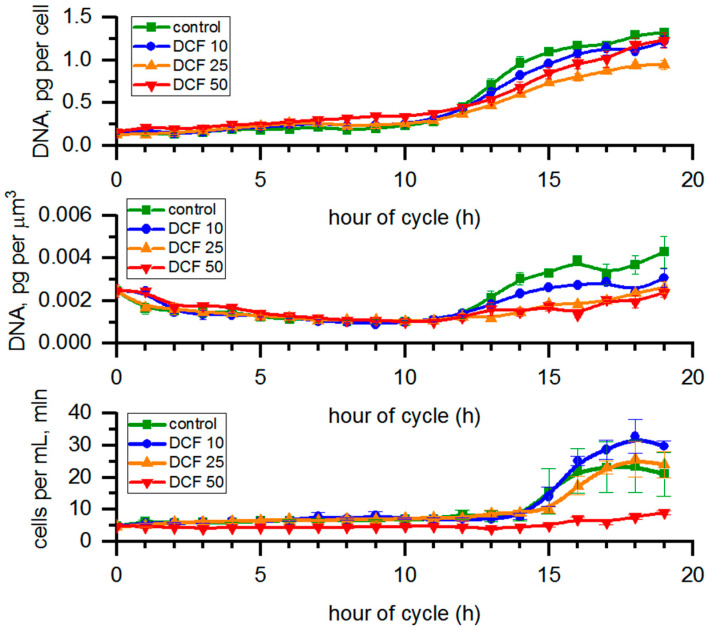
DNA content calculated per single mother or µm^3^ of total cell volume in sample and the number of cells per mL. Data are presented as means ± SE. Cell number of DCF50 and DNA content per µm^3^ of all DCF groups were statistically significantly different from controls (correlation comparison, *p* < 0.05, *n* = 3). Data of DNA content per cell are presented as pg per mother cells even at the time of cell division to allow simple comparison across the groups.

A significant increase in the doubling time in all DCF-treated populations of *C. reinhardtii* (Table 1) was in line with the analysis showing an increase for the DNA content in cells (Figure 5), which tended to be delayed in all DCF-treated populations since there were differences in the cell volumes among individual groups. The DNA content normalized per cell volume was used to visualize if the process of DNA replication (and the connected process of cell division) were related to reaching a certain cell size within the individual groups. The results of DNA content per µm^3^ of total biomass volume in a sample confirmed a statistically significant delay in DNA replication for all three treatment groups. In DCF50, an increase in DNA content was observed at earlier hours of the cell cycle (before replication of nuclear DNA started). A similar increase was also present in the early hours of the cell cycle in DCF10 and DCF25 (between fifth and ninth hours of cycle).

The analysis of nuclear divisions revealed no significant differences between DCF-treated cells as compared to control (Figure S2).

## 4. Discussion

Data described in our previous studies showed that in asynchronous cultures of *C. reinhardtii,* DCF caused oxidative stress and affected photosynthesis [9,35,36]. However, it was not clear what was its effect would be on growth and cell reproduction (cell cycle) and if they were both affected similarly or not. It is known that growth is less sensitive to potentially toxic compounds than cell reproduction is [20]. Thus, treatment of *C. reinhardtii* with different concentrations of DCF might have different effects on cell growth and on cell cycle progression. In searching for the concentration that had little-to-no effect on growth but affected cell cycle progression, we examined four different DCF concentrations corresponding to the toxicological values of EC10, EC25, EC50 and EC75 (here described as DCF10, DCF25, DCF50 and DCF75) on synchronized cultures of *C. reinhardtii*. All the concentrations of DCF used in our study were higher than the environmentally relevant concentrations ranging from 1 ng/L to 1 mg/L [37,38,39]. Yet, the cultures of *C. reinhardtii* were able to cope with all the concentrations except the highest one, DCF75, for at least 24 h. In our previous study, the asynchronous cells were able to cope with even higher DCF concentrations for 24 h [36]. In our experiments, DCF75 caused a strong cytotoxic effect, lysis, in which about 70% of cells died during the first hours of the cell cycle. Since the primary purpose of our study was to discriminate the effect of DCF on growth and cell division, this DCF concentration was deemed too high and detrimental, so it was not used in further experiments.

Interestingly, similar immediate DCF toxic effects were observed within 1 h of the DCF50 culture illumination, which resulted in about a 30% drop in cell numbers (Figure 4). It may be assumed that some daughter cells that consumed their energy storage reserves during the dark phase were unable to cope with the combination of light stress combined with the impact of DCF50, possibly due to its toxic effect on young chloroplasts and oxidative stress generation [9,36]. The above assumption is supported by the observation that immediately after th dark/light transition, the overall efficiency of photosynthesis (performance index, quantum yield) transiently dropped in control cells, and this effect was enhanced by DCF50 (Figure 3). However, the surviving cells continued their growth in a similar manner to the control cells until the end of the cell cycle. Despite this, chl *a* and carotenoid content in DCF50 cells was significantly lower, particularly at the end of the cell cycle (Figure 2), which can be regarded as symptomatic of oxidative damage [40]. Yet, the DCF50 influence on photosynthesis was only detectable for a single parameter, ψ0 (Figure 3). 

Furthermore, DCF50 cells contained significantly more starch (Figure 1), a trend which was again more pronounced when DNA replication, nuclear division, and cell division were being performed in the control culture (Figure 5). The starch content is known to be in balance between the material and energy supply provided by photosynthesis and their consumption [41]. Cell reproduction was established as the main starch (and energy) consumer both under light and in dark conditions [42,43], so the larger starch content could have been caused by a delay and less cell division in DCF50 culture (Figure 1 and Figure 5). In light of the above, it could be supposed that significant amounts of DCF reached the cell interior within a couple of hours of the incubation, and the effect was prolonged. The speed of DCF entry and action is also supported by the observed effect on CP attainment that was analyzed in dark in the absence of DCF but showed significant differences between the DCF50 variant and control (Figure 5). Similar behavior was reported by Fu et al. (2017) [44] for suspensions of *Arabidopsis thaliana* (Magnoliophyta) cells.

The use of synchronous cultures allowed us to discriminate the effect of DCF on growth and cell cycle progression. In DCF25 and DCF10 cultures, no significant changes in cell growth were detected (Figure 1) until the 14th to 15th hour of the cell cycle when daughter cells began to be released. Furthermore, the total RNA and protein content in cells was similar in the treated and control cells, confirming that DCF did not affect individual cell growth (Figure 1). Interestingly, a significant difference was detected for RNA and protein in DCF50 variant (and for RNA in DCF25) when the biomolecule content was normalized per culture volume (Figure S1). This suggested that cell composition of DCF50 changed, probably in the advantage of increased starch content. In DCF25 cultures, there was a trend of chl *a* and carotenoid decrease and a starch increase (Figure 1) similar to that of the DCF50 cells but to a lesser extent. Only the carotenoid content was statistically significantly different from the control. In DCF10 cultures all the physiological and biochemical parameters, except of the photosynthesis ones, remained close to the control level as expected (Figure 1, Figure 2, Figure 3 and Figure 5). However, under this DCF concentration, some symptoms of toxicant acclimation could be seen. First, during later hours of cell cycle, there was an increase in carotenoid content in the DCF10 cells (Figure 2), which might suggest that tolerance mechanisms were induced in the progeny cells [45] (Figure 2). This hypothesis was supported by the work of Lemoine and Schoefs (2010) [40], who reported that in plants and algae, carotenoid synthesis and accumulation is the cell’s response to stress factors.

As the above-mentioned observations confirm that DCF alters the cell cycle progression rather than single cell growth [36], we focused on cell cycle-related parameters. As mentioned above, DCF10 and DCF25 concentrations did not affect individual growth of *C. reinhardtii* cells (Figure 1). Due to similar growth, the ability of cells to reach critical size and attain CPs was comparable among control and DCF10 and DCF25 (Figure 5). Interestingly, cells within the population attained the first and second CPs slightly faster than the control did. That tendency was more pronounced after 50% of the cells had attained the first and second CPs, and was also valid for the third. An opposite trend was observed for DCF50, where not only was CP attainment significantly slower, but only approx. 65% of cells attained the first and 50% attained the second CP compared to 100% in the control. Thus, there was a clear-cut difference between the effect of different DCF concentrations on *C. reinhardtii* cells. Moreover, it should be kept in mind that CP attainment is assessed as the ability of the cell to complete cell division in the dark. Since DCF at high concentration affects cell division, the CP attainment test may not adequately reflect real CP attainment. However, since the CP test was performed on a solid medium without DCF, cell division impairment was most likely caused by DCF that had accumulated during cell growth in light conditions that then subsequently affected cell division. Characteristics of the CP curves (for the first and second commitment) in DCF50 implied that toxic effects for cell division occurred very shortly and irreversibly after DCF treatment. Thus, DCF accumulated in the cell, then later had an impact on the first and second cell divisions. No dilution of DCF from cell into the solid medium is suggested as the first and second division were affected similarly.

An analysis of cell division showed that the first daughter cells were released after the 10th hour of the cell cycle in the control, DCF10 and DCF25 and with a 1 h delay in DCF 50 where the first daughter cells appeared at the 12th hour. Moreover, the midpoints of division were delayed by approximately 1 h in DCF10 and DCF25 compared to controls. In DCF50, less than 40% of the culture divided, so the midpoint was never reached. A comparison of mother-cell volume at the end of the cell cycle showed that in control and DCF10, the majority of daughter cells was released at or after the 15th hour. However, in DCF25 it happened with a 1 h delay at the 16th hour, while in DCF50 we observed a 4 h delay when compared to the controls at the 19th hour. 

Since the cells were cultivated on light and growth was not significantly affected, the DCF50-treated cells divided at significantly higher volumes of mother cells compared to the control yet they produced smaller daughter cells (Table 1). This may suggest that DCF50 affected the release of daughter cells at the end of cell division and that these daughter cells grew inside the mother cell capsule before being released. This seems to be the case as divided daughter cells within the mother cell wall were observed as soon as 10 h into the cell cycle, yet they were not released until much later. This inability to release daughter cells from mother cell wall in DCF50 can be considered as the exhibition of a kind of stress phenotype by *C. reinhardtii* caused by DCF leading to a temporary palmelloid state [46]. Furthermore, in all the variants, the total number of daughter cells produced (division number) significantly decreased compared to the controls suggesting that even when the entire population divided as in DCF10 and DCF25, the mother cells divided into fewer daughter cells. Such cells were only slightly (DCF10), or significantly (DCF25) larger than the control cells (Table 1).

Cell number analysis displayed a lower number of daughter cells at the end of the cell cycle in the DCF10 and DCF25 groups compared to the control. Furthermore, cell division took longer as the doubling time of both variants increased by 21% compared to the control (Figure 4 and Table 1). In the DCF50 group, not only a lower number of daughter cells at the end of the cell cycle were observed but the number of the dividing cells within the population was significantly lower (Figure 4, Table 1). In fact, cell division only occurred in around 30% of the population surviving after 30% of the cells had died within the first hour after illumination. Furthermore, in the DCF50 group, the doubling time was even higher than for other DCF concentrations (we excluded the 30% of dead cells from further analysis), indicating impairment in cell division caused by DCF.

## 5. Conclusions

**Figure 6:**
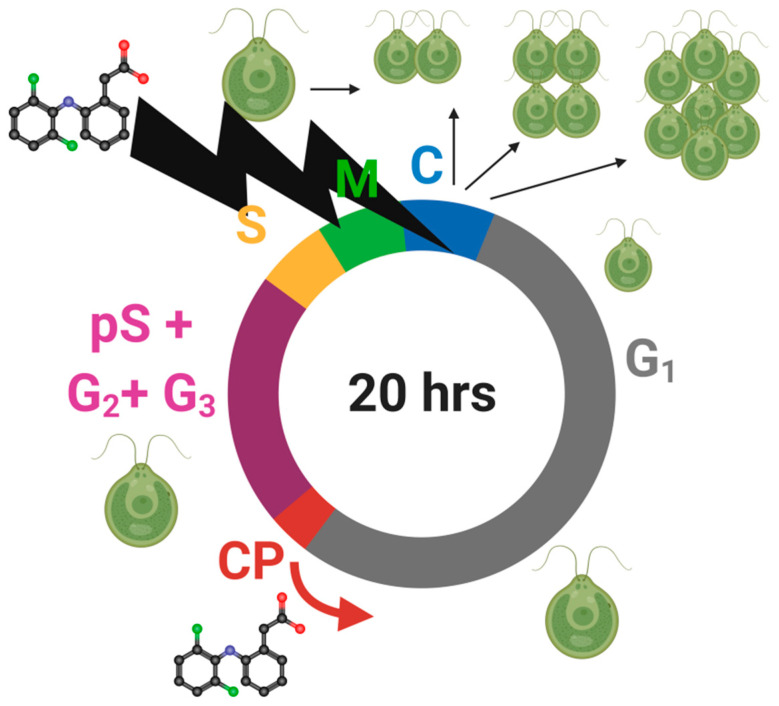
Illustration of the DCF influence on *Chlamydomonas reinhardtii* cell cycle (scheme of cell cycle by Zachleder et al. [48], modified).

The use of synchronous cell cultures allowed us to verify the hypothesis that one of the causes of DCF phytotoxicity was the disorder of cell cycle progression, and extend the findings of our previous works [9,35,36]. The anti-proliferative effect of DCF, reported in the literature for mammalian cells [47], also appeared to be valid for *C. reinhardtii* cells. DCF treatment did not influence individual cell growth or the attainment of CPs in lower concentrations of DCF. At higher DCF concentrations, CP attainment was delayed. For all DCF-treated groups, we observed a DCF-dependent delay in DNA replication, delay in cell division and a decreased number of daughter cells. For a more comprehensive view of the effects of DCF influence on *C. reinhardtii* cell cycle, see the model of DCF action (Figure 6).

## Figures and Tables

**Table 1:** The population doubling time (*Td*), the cells division number (DN), the mode volume of mother (V_MC_) and daughter (V_DC_) cells. Data are presented as means ± SE.

	*Td* (h)	DN	V_MC_ (µm^3^)	V_DC_ (µm^3^)
control	10.42 ± 0.40	6.20 ± 0.44	461.30 ± 23.77	39.88 ± 2.64
DCF10	12.61 * ± 0.54	4.51 * ± 0.26	505.87 ± 55.46	44.62 ± 2.80
DCF25	12.70 * ± 0.25	4.46 * ± 0.13	491.83 ± 31.67	51.20 * ± 3.86
DCF50	16.08 * ± 1.16	3.26 * ± 0.30	616.20 * ± 10.45	32.06 ± 6.90

* indicates statistically significant differences between control and treated cells at *p* < 0.05 (Mann–Whitney U test, *n* = 3).

## Data Availability

All data presented in this study are available within this article or Supplementary Materials. There are no special databases associated with this manuscript.

## Supplementary Materials

The following are available online at https://www.mdpi.com/article/10.3390/cells10081936/s1, Figure S1: The RNA, protein and starch content per µm^3^ of total volume of cells in culture. Data represent means ± SE. RNA content per µm3 for DCF25 and DCF50 and protein content per µm3 for DCF50 was statistically different from controls. The starch content was not significant for any of the samples (correlation comparison, *p* < 0.05, *n* = 3). Figure S2: The photomicrograph of cells after 11 hours of the experiment with divided nuclei after DAPI staining (40×). A—control, B—DCF 10, C—DCF25, D—DCF50.
