# Supplementary material for: Diclofenac Alters the Cell Cycle Progression of the Green Alga Chlamydomonas reinhardtii
Source: Cells. 2021 Jul 30;10(8):1936. doi: 10.3390/cells10081936 (PMC8392695; doi:10.3390/cells10081936)
Supplement: Supplementary file 1 [file cells-10-01936-s001.zip › cells-1299385-supplementary.pdf]

Supplementary Materials:

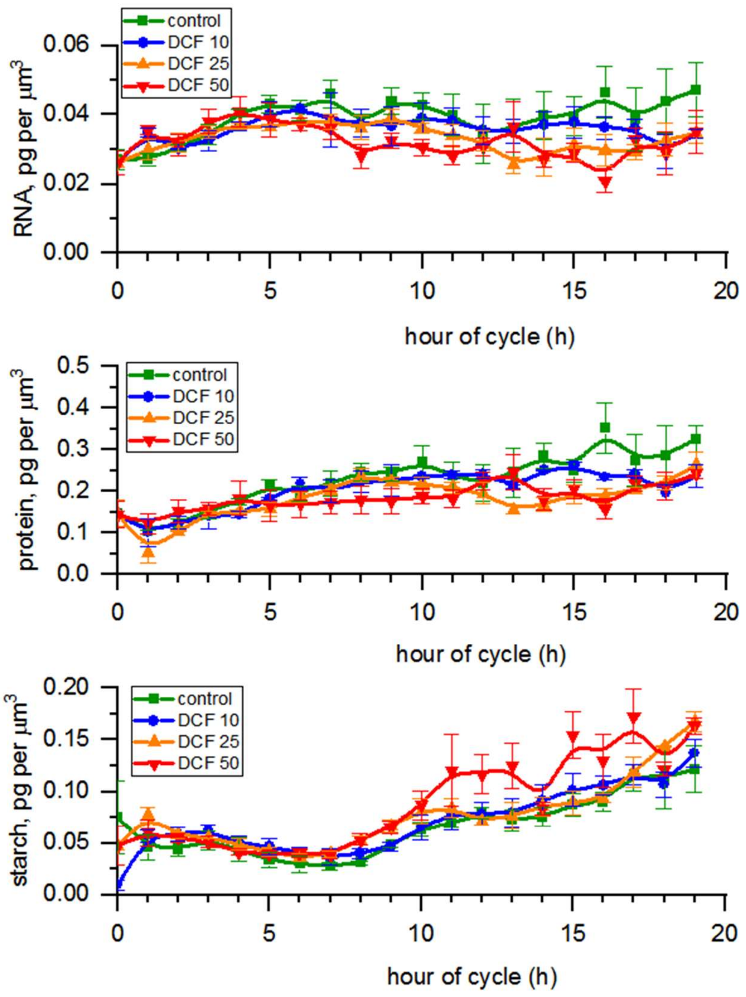

Figure S1. The RNA, protein and starch content per μm^3 of total volume of cells in culture. Data represent means ± SE. RNA content per μm^3 for DCF25 and DCF50 and protein content per μm^3 for DCF50 was statistically different from controls. The starch content was not significant for any of the samples (correlation comparison, $p < 0.05$, $n = 3$).

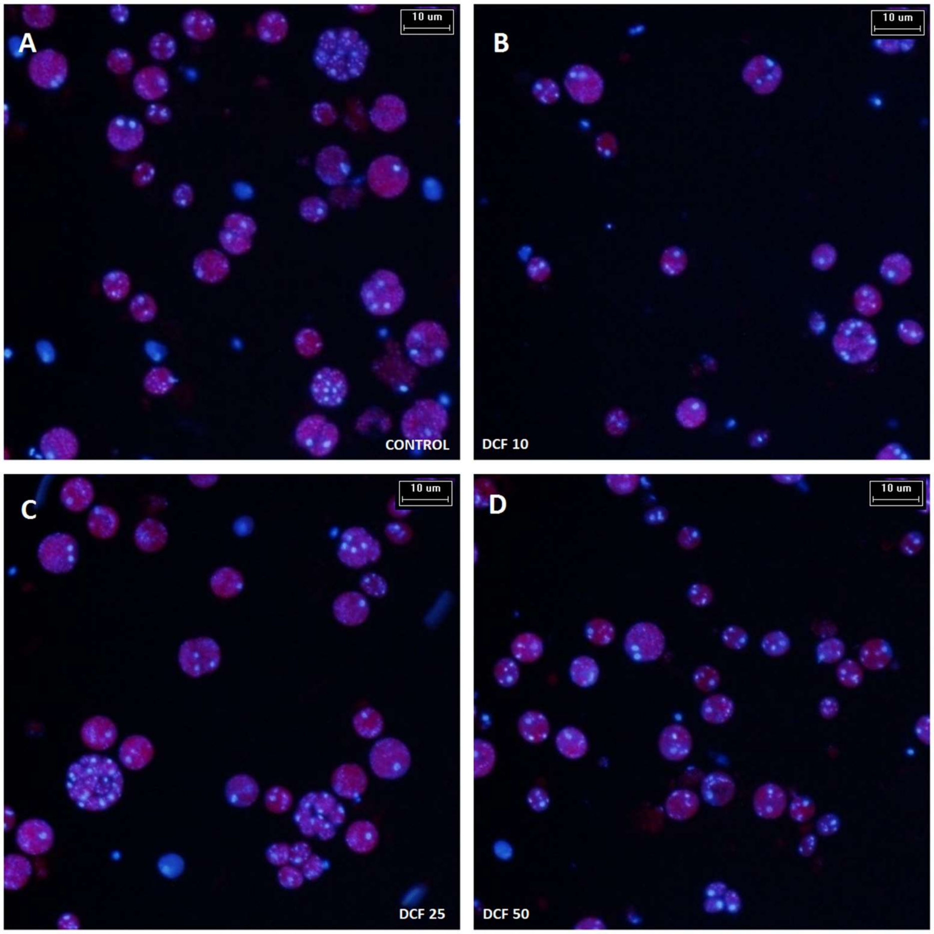

Figure S2. The photomicrograph of cells after 11 hours of the experiment with divided nuclei after DAPI staining (40x). A – control, B – DCF 10, C- DCF25, D- DCF50.
